# Bacteremia during dacryocystorhinostomy: results of intra-operative blood cultures

**DOI:** 10.1186/s12348-014-0027-7

**Published:** 2014-10-09

**Authors:** Mohammad Javed Ali, Anuradha Ayyar, Swapna R Motukupally, Savitri Sharma, Milind N Naik

**Affiliations:** 1 Dacryology Service, L.V.Prasad Eye Institute, Hyderabad 34, India; 2 Jhaveri Microbiology Center, L.V.Prasad Eye Institute, Hyderabad 34, India

**Keywords:** Dacryocystorhinostomy, Bacteremia, Blood culture, Antibiotics

## Abstract

**Background:**

The aims of the study are to assess the prevalence of bacteremia during dacryocystorhinostomy (DCR) and to assess whether there is a need for post-operative prophylaxis. Prospective interventional study of 52 consecutive dacryocystorhinostomy performed in 50 patients over a period of 1 year from 2013 to 2014. Blood was drawn under strict aseptic conditions during two separate time points: fashioning of the nasal mucosal and creation of lacrimal sac flaps. The blood was inoculated into two blood culture bottles: the dual media as well as Columbia broth. Following withdrawal of blood, all patients received an intraoperative single dose of a cephalosporin antibiotic. Clean cases of primary acquired nasolacrimal duct obstructions (PANDO) without any sac discharge upon marsupialization (22%, 11/50) were not prescribed routine post-operative prophylaxis, whereas the remaining were prescribed oral antibiotics for 5 days.

**Results:**

The mean age of patients was 41 years (range, 4–61 years). The most common diagnosis (70%, 35/50) was primary acquired nasolacrimal duct obstruction. Acute dacryocystitis was noted in 12% (6/50). External DCR was performed in 65% (34/52) and endoscopic DCR in 35% (18/52) of the cases. All the blood cultures were uniformly negative both in terms of abnormal physical changes in media as well subcultures; 22% (11/50) did not receive post-operative antibiotic prophylaxis. None of the patients developed any signs of wound infections. The anatomical and functional success rate was achieved in 98%.

**Conclusions:**

This study did not find any intraoperative bacteremia during dacryocystorhinostomy and that none had wound infection irrespective of post-operative prophylaxis.

## Background

Dacryocystorhinostomy (DCR) is a commonly performed procedure for managing nasolacrimal duct obstructions [1],[2]. Bacteremia is the presence of viable bacteria in the blood stream. Since blood is a sterile environment, bacteremia is always abnormal. Bacteremia has been reported during surgery, especially when involving mucous membranes such as that of the oral mucosa and the lower gastrointestinal tract [3]. The incidence of bacteremia during probing for congenital nasolacrimal duct obstruction has been reported in up to 17.5% of patients [4]–[6]. In patients with infant dacryocystitis, a bacteremia prevalence of 22.5% (*n* = 22) has been documented [7]. Venugopalan et al. reported bacteremia rates of 4% in extra ocular surgeries [6]. Only one patient of DCR (*n* = 6) in their series demonstrated a bacteremia with *Haemophilus influenzae*.

Although DCR surgery can be classified as a clean contaminated type of operative procedure [8], the reported rates of post-operative cellulitis following open lacrimal surgeries were as high as up to 18% in patients without post-operative antibiotic prophylaxis [9],[10]. However, other studies have documented contrary results and argued against routine prophylaxis [11],[12]. Hence, there are important and unresolved issues regarding post-operative antibiotic prophylaxis and its role in the prevention of wound infection. To the best of our knowledge, there has been no study that focused on DCR-associated bacteremia. The current study reports the results of intraoperative blood cultures during a DCR and their clinical profiles and outcomes.

## Methods

Prospective interventional study of 52 consecutive dacryocystorhinostomy, performed in 50 patients over a period of 1 year from 2013 to 2014. Institutional review board approval was obtained. Blood (10 ml) was drawn under strict aseptic conditions from antecubital fossa through a venipuncture. This was uniformly performed during two time points: fashioning of the nasal mucosal and during creation of lacrimal sac flaps. The top of the culture bottle, which is protected by a sterile cap is removed and the vials immediately inoculated with blood using a separate sterile needle. Following withdrawal of blood, all patients received an intraoperative single dose of a cephalosporin antibiotic (Cefuroxime). All acute dacryocystitis cases were operated by an endoscopic approach under general anesthesia. All patients were intubated, and stents were removed at 6 weeks. Clean cases of primary acquired nasolacrimal duct obstructions (PANDO) without any sac discharge upon marsupialization (22%, 11/50) were not prescribed routine post-operative prophylaxis, whereas the remaining were prescribed oral antibiotics for 5 days. Post-operative endoscopic assessments were performed at day 1 and weeks 1, 6, and 12.

###  Blood culture protocol

**Figure 1:**
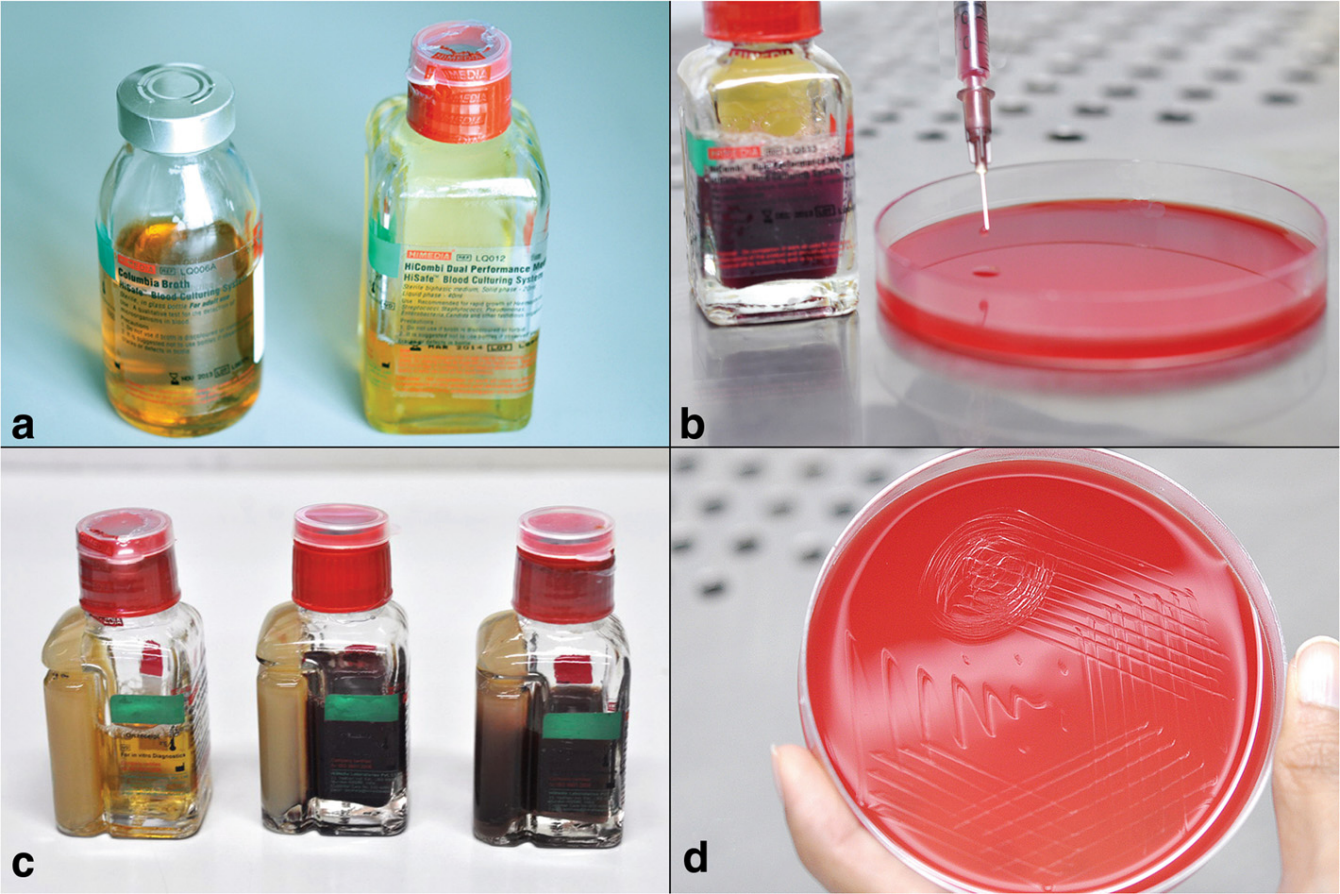
**Blood culture protocol.** Photograph of blood culture media showing liquid Columbia broth to the left and the dual medium to the right **(a)**. Venting procedure in a biological safety cabinet. Note the subculture inoculation on to 5% sheep blood agar **(b)**. Dual medium blood culture bottle (from left to right) before inoculation, day 7 and day 14. Note the absence of turbidity **(c)**. Inoculated blood agar showing absence of growth from subcultures **(d)**.

Two blood culture media (Himedia Laboratories, Mumbai, India) were used (Figure 1a). The first was a dual media (20 ml solid agar coated surface and 40 ml liquid medium), and the second was Columbia broth (70 ml) (Figure 1a). The dual media are rich in growth factors that enable detection of both aerobic and anaerobic organisms where as Columbia broth facilitates rapid growth of fastidious organisms. The inoculated vials were immediately placed in blood culture machines for incubation at 37°C and observed daily for visible turbidity, color change, and hemolysis in the medium. Venting was carried out in a biological safety cabinet (Figure 1b). The protocol in cases of any change in the media was to subculture it onto 5% sheep blood agar and chocolate agar plates, incubate at 37°C and observe for growth up to 48 h. In all cases where the media did not produce any turbidity, subcultures were done on day 7 and day 14 onto 5% sheep blood agar and chocolate agar plates and incubated for 48 h (Figure 1b). In case of positive growth on blood agar, the protocol was designed to process the isolate for identification and antibiotic susceptibility testing.

## Results

The mean age of patients was 41 years (range, 4–61 years). The male/female ratio was 1:2.8 (13:37). The most common diagnosis (70%, 35/50) was PANDO. Other diagnoses include acute dacryocystitis (12%, 6/50), failed external DCR (8%, 4/50), traumatic nasolacrimal duct obstruction (6%, 3/50), and persistent congenital nasolacrimal duct obstructions (4%, 2/50). External DCR was performed in 65% (34/52) and endoscopic DCR in 35% (18/52) of the cases. All the blood cultures were uniformly negative both in terms of abnormal physical changes in media (Figure 1c) as well subcultures (Figure 1d); 22% (11/50) did not receive post-operative antibiotic prophylaxis. None of the patients developed any signs of wound infections. At a minimum follow-up of 3 months post-stent removal, one patient developed anatomical failure secondary to cicatricial closure of the ostium. This patient had received post-operative antibiotic prophylaxis. The anatomical and functional success rate was achieved in 98%.

## Discussion

This study showed no evidence of intraoperative bacteremia during a dacryocystorhinostomy. The subset of patients who did not receive post-operative antibiotic prophylaxis failed to show any evidence of post-operative infections. Although the sample size was small, no correlation between antibiotic usage and success was noted.

One of the most significant milestones in the history of medicine is the introduction and usage of antibiotics. Almost half of the prescriptions are reported to be used prophylactically [13]. Although, there is a decrease trend in the use of post-operative prophylaxis, the practice is still widely prevalent in the developing world.

Eippert et al. documented probing-induced bacteremia in 17.5% of their patients (*n* = 40) and recommended antibiotic prophylaxis especially in those at risk of infective endocarditis [4]. Grech et al. supported this argument [5]. Baskin et al. [7] reported bacteremia in 22.5% of their patients with infant dacryocystitis and recommended prophylaxis. However, contrary opinions were published by Venugopalan et al. [6] and Pollard [14], where stringent preoperative aseptic precautions were considered enough [6] and no untoward effects without antibiotic prophylaxis was demonstrated even in neonatal acute dacryocystitis [14].

Walland and Rose demonstrated a fivefold reduction in the incidence of soft tissue infections in their DCR patients (*n* = 280) with post-operative antibiotic prophylaxis [9]. Vardy and Rose subsequently showed more than tenfold decrease in infective cellulitis with prophylaxis in their DCR series (*n* = 265) [10]. They showed comparable efficacy between intraoperative and post-operative prophylaxis. However, opinions contrary to these are well established. Pinar-Sueiro et al. [11] in their very large series of 697 external dacryocystorhinostomy questioned the routine use of prophylactic antibiotics. They demonstrated that routine use of antibiotics failed to lower the rate of post-operative infections but suggested that they may play a role in high-risk cases like acute dacryocystitis and mucopyocele. Dulku et al. [12] further elaborated that for routine prophylaxis to prevent one DCR infection, the number needed to treat would be 104 and suggested that such routine prophylaxis is hard to justify.

## Conclusions

In conclusion, the current study neither demonstrated any bacteremia in any of the cases including acute dacryocystitis nor any post-operative infections in patients without antibiotic prophylaxis. Intraoperative single dose of antibiotic was noted to be sufficient. If the additional potential side effects of the drugs and the rising menace of antibiotic resistance are taken into consideration, we suggest that the routine use of post-operative prophylactic antibiotics may not be justified.

### Ethics statement

This study has been reviewed by the ethics committee and has been performed in accordance with the ethical standards laid down in the 1964 Declaration of Helsinki. Informed consent was obtained from the patients.

## Abbreviations

DCR: dacryocystorhinostomy

PANDO: primary acquired nasolacrimal duct obstruction

## Competing interests

The authors declare that they have no competing interests.

## Authors’ contributions

MJA contributed to the concepts and drafted the manuscript. AA was involved with data collection and analysis. SRM and SS contributed to the microbiological analysis, and MNN performed review of the manuscript. All authors read and approved the final manuscript.
